# Choosing an imbalance metric for covariate-constrained randomization in multiple-arm cluster-randomized trials

**DOI:** 10.1186/s13063-019-3324-5

**Published:** 2019-05-28

**Authors:** Jody D. Ciolino, Alicia Diebold, Jessica K. Jensen, Gerald W. Rouleau, Kimberly K. Koloms, Darius Tandon

**Affiliations:** 1 Department of Preventive Medicine, Division of Biostatistics, Feinberg School of Medicine, Northwestern University, 680 N Lake Shore Drive, Suite 1400, Chicago, IL 60611 USA; 2 Center for Community Health, Institute for Public Health and Medicine, Feinberg School of Medicine, Northwestern University, Chicago, IL USA; 3 College of Medicine, University of Cincinnati, Cincinnati, OH USA; 4 Hill-Rom Inc., Chicago, IL USA

**Keywords:** Cluster randomization, Covariate-constrained randomization, Cluster-randomized controlled trial, Continuous covariate, Imbalance

## Abstract

**Background:**

In cluster-randomized controlled trials (C-RCTs), covariate-constrained randomization (CCR) methods efficiently control imbalance in multiple baseline cluster-level variables, but the choice of imbalance metric to define the subset of “adequately balanced” possible allocation schemes for C-RCTs involving more than two arms and continuous variables is unclear. In an ongoing three-armed C-RCT, we chose the min(three Kruskal–Wallis [KW] test *P* values) > 0.30 as our metric. We use simulation studies to explore the performance of this and other metrics of baseline variable imbalance in CCR.

**Methods:**

We simulated three continuous variables across three arms under varying allocation ratios and assumptions. We compared the performance of min(analysis of variance [ANOVA] *P* value) > 0.30, min(KW *P* value) > 0.30, multivariate analysis of variance (MANOVA) *P* value > 0.30, min(nine possible *t* test *P* values) > 0.30, and min(Wilcoxon rank-sum [WRS] *P* values) > 0.30.

**Results:**

Pairwise comparison metrics (*t* test and WRS) tended to be the most conservative, providing the smallest subset of allocation schemes (10%–13%) meeting criteria for acceptable balance. Sensitivity of the min(*t* test *P* values) > 0.30 for detecting non-trivial imbalance was 100% for both hypothetical and resampled simulation scenarios. The KW criterion maintained higher sensitivity than both the MANOVA and ANOVA criteria (89% to over 99%) but was not as sensitive as pairwise criteria.

**Conclusions:**

Our criterion, the KW *P* value > 0.30, to signify “acceptable” balance was not the most conservative, but it appropriately identified imbalance in the majority of simulations. Since all are related, CCR algorithms involving any of these imbalance metrics for continuous baseline variables will ensure robust simultaneous control over multiple continuous baseline variables, but we recommend care in determining the threshold of “acceptable” levels of (im)balance.

**Trial registration:**

This trial is registered on ClinicalTrials.gov (initial post: December 1, 2016; identifier: NCT02979444).

**Electronic supplementary material:**

The online version of this article (10.1186/s13063-019-3324-5) contains supplementary material, which is available to authorized users.

## Background

In cluster-randomized controlled trials (C-RCTs), the clustered nature of intervention and data present added statistical and logistical complexities, suggesting the requirement for even more meticulous planning than a typical individually randomized clinical trial [1–3]. In C-RCTs, as in individually randomized clinical trials, randomization algorithms that control imbalance in baseline prognostic factors are ideal [4–6], but the complexities are heightened in C-RCTs as the unit of randomization is a cluster (usually an entire site) rather than an individual participant. In general, imbalance in influential baseline variables across arms, whether in individually randomized [7–9] or cluster-randomized trials [1, 2, 4–6], has the potential to result in biased treatment effect estimates and may decrease precision on intervention effect estimates. In a recent review, Ivers et al. [4] summarize methods of restricted randomization for controlling cluster-level baseline variable imbalance and specifically address advantages and limitations of each. Common methods include matching, stratification, minimization, and covariate-constrained randomization (CCR) [4].

Since it can efficiently control imbalance in multiple baseline variables simultaneously, multiple authors [1, 2, 4–6, 10–13] recommend CCR, assuming its logistical feasibility for a given trial over other methods (i.e., simple randomization and stratified randomization). Although there are many variations [6, 10–13], the general algorithm suggests the following:

1. Enumerate all or a large subset (e.g., 100,000) of possible allocation schemes.
2. Evaluate “imbalance” for each iteration.
3. According to a pre-defined criterion (e.g., the lower 10% of the imbalance metric’s empirical distribution), define a subset of “acceptable” allocations.
4. Randomly select one of these acceptable allocations for implementation.

Several authors [6, 10–13] have proposed the choice of the imbalance metric (step 2 above) but these measures often assume that baseline variables of interest are categorical [10, 11, 13] or that the C-RCT involves two study arms [6, 12] or both. In handling continuous baseline variables in a CCR algorithm, Raab and Butcher [6] suggest a weighted sum of mean differences across arms, squared (i.e., “B” or “B_(l2)_”), whereas Li et al. [12] propose a weighted sum of absolute mean differences across arms (denoted “B_(l1)_”). Common practice involves the use of the lower 10th percentile of these metrics to define the pool of “acceptable” allocation schemes [6]. These proposed metrics, however, may not readily extend to C-RCTs with more than two study arms, and the notion of the 10th percentile of the distribution of these abstract metrics may carry little meaning in general. That is, the researcher may be left wondering whether the randomization algorithm employed truly achieved comparable arms in his or her C-RCT.

In this article, we present a complex three-arm C-RCT case study that involved CCR, aiming to control imbalance for three continuous baseline variables. This case demonstrated two gaps in the literature regarding CCR: minimal guidance for (a) choosing an imbalance metric that readily extends to more than two arms for continuous variables and (b) defining an intuitive threshold of imbalance to ensure adequate balance in the pool of possible schemes for implementation. Here, we propose an imbalance metric—the minimum Kruskal–Wallis (KW) test *P* value comparing variables across arms—and a corresponding threshold of acceptability (*P* >0.30) to guide similar C-RCT randomization.

In the sections that follow, we present the case study and randomization methods implemented for the specific study, a series of simulation studies exploring the performance of the proposed metric in comparison with others, and, finally, overall conclusions and recommendations based on our findings. It is important to note that the simulations presented illustrate a series of hypothetical trials inspired by the case study.

## The Mothers and Babies Case Study

The Patient-Centered Outcomes Research Institute (PCORI)-funded study “Comparing the Effectiveness of Clinicians and Paraprofessionals to Reduce Disparities in Perinatal Depression” is a C-RCT randomly assigning 42 home visitor (HV) sites in the Midwest region of the US to one of three arms (contract number: AD-1507-31,473). Previously, the investigators of this study established the efficacy of the Mothers and Babies (MB) Course when augmenting core HV services in preventing onset of postpartum depression and reducing depressive symptoms when led by mental health (MH) professionals [14, 15]. However, to date, there are no interventions led by non-health or non-MH professionals that have demonstrated efficacy in preventing the onset and worsening of postpartum depression among low-income women. Thus, we planned a C-RCT in which HV clients receive (a) MB delivered by MH professionals, (b) MB delivered by paraprofessional HVs, or (c) usual HV services. This study design allows the conduct of a superiority trial that compares the efficacy of MB delivered by paraprofessional HVs versus usual care, and the design also allows a non-inferiority analysis that compares the effectiveness of MB delivered by MH professionals versus paraprofessional HVs. Should this study find that paraprofessional HVs are not inferior to MH professionals in delivering the intervention, HV programs throughout the US could implement MB with paraprofessional HVs—an approach that is considerably more efficient and cost-effective than employing MH professionals.

The study plan employed a modified CCR design at the site level by using unequal allocation: for every one control site, we enrolled three MH-led sites and three HV-led sites (i.e., 1:3:3 allocation for control: MH delivery of MB: HV delivery of MB). We intended to control imbalance at the site level at baseline in three pre-specified potential covariates: (1) percent minority (i.e., non-White) clients as reported by the site, (2) site-reported yearly client volume, and (3) population density of site location area, defined by site zip code. We treated all three variables as continuous for randomization purposes, as categorizing across three arms with unequal allocation will inevitably result in low cell counts and loss of efficiency. With minimal guidance from the literature and experience with regard to choice of imbalance metric, we chose to use what we deemed an intuitive measure of imbalance: the KW test across the three arms for each of the three variables. We employed the following general randomization algorithm for this study. It is worth noting that there were added complexities regarding “waves” of randomization and adaptations for dropouts, but for the sake of simplicity, the general logic is below:

1. Enumerate a large number of possible allocation schemes (100,000), each with the planned 1:3:3 allocation ratio.
2. For each possible scheme, calculate three KW test statistics and corresponding *P* values comparing rank values across the three arms (for each variable: percent minority, yearly volume, and population density).
3. If min(KW test *P* values) > 0.30 [9], then accept the iteration into a pool of possible scenarios.
4. Randomly select one of these schemes meeting criteria in step 3 as the one chosen for implementation in the present study.

**Fig. 1:**
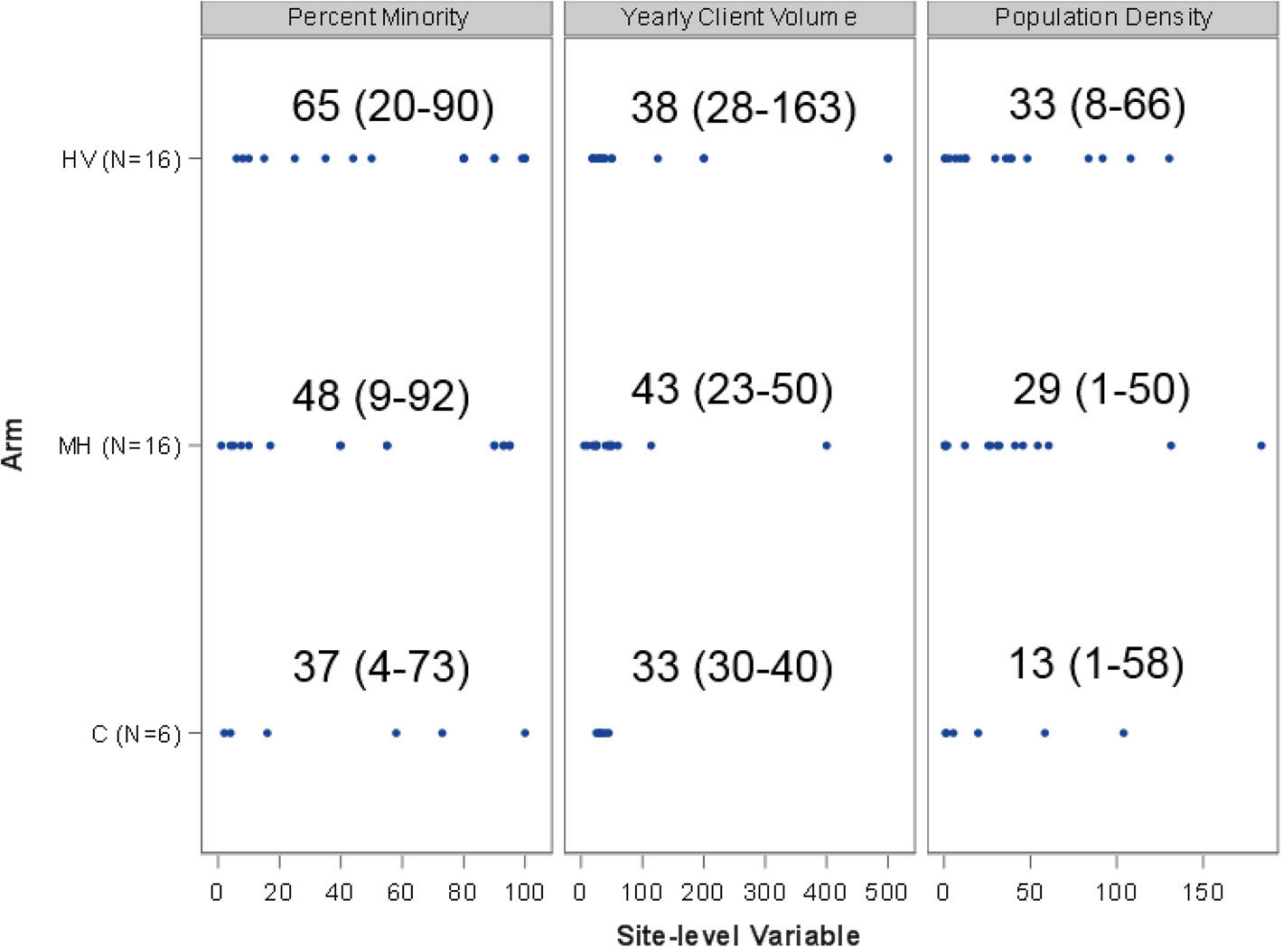
Distribution of site-level randomization variables by arm for trial NCT02979444. We planned for a total of 42 randomized sites (6:18:18), but owing to dropout we have 38 active sites. Sample size and power considerations accounted for dropout that we observed. Medians (interquartile ranges) are displayed in each arm for each variable above

This agreed-upon randomization algorithm required upfront data collection from each site, careful but straightforward programming, and less than a 24-h computing lag time to run required iteration scenarios on a local computer. We deemed this method of randomization intuitive and rather simple to implement. The question remains, however, whether our randomization “worked” or achieved imbalance control in these variables. Figure 1 illustrates the resultant distribution of each variable and relevant summary statistics for sites randomly assigned to date.

Although the distributions appear well balanced, the common question “Did the randomization method work?” came about. To explore the performance of this algorithm and imbalance metric, we performed a simulation study comparing the chosen metric of imbalance with other potential metrics of imbalance which we may have chosen. We aimed to (a) explore which of a given list of imbalance metrics tended to be most conservative overall and (b) determine which imbalance metrics adequately detected covariate-level imbalance. The following section provides details of simulation methods, exploring several metrics of imbalance. The simulation logic and metrics explored emulate those of a similar exploration by Ciolino et al. [7] in the individually randomized trial setting. In this context, the term “conservative” refers to a metric that is less likely to accept an individual allocation scenario into the pool of possible scenarios (i.e., one that is more restrictive or constrained).

## Methods

We conducted multiple simulation studies inspired by this case study: one from hypothetical data under several different scenarios meant to mirror potential real-world data and one resampling actual data from the case study. The basic simulation logic is as follows:

1. Assume that three cluster-level variables (X1, X2, and X3) come from a multivariate normal distribution with some pre-specified level of correlation.
  - $$ {\displaystyle \begin{array}{c}{X}_1\\ {}{X}_2\\ {}{X}_3\end{array}}\sim MVN\left(\begin{array}{c}{\mu}_1\\ {}{\mu}_2\\ {}{\mu}_3\end{array},\begin{array}{ccc}{\sigma}_1& {\sigma}_{12}& {\sigma}_{13}\\ {}{\sigma}_{12}& {\sigma}_2& {\sigma}_{23}\\ {}{\sigma}_{13}& {\sigma}_{23}& {\sigma}_3\end{array}\right)= MVN\left(\boldsymbol{\mu}, \boldsymbol{\Sigma} \right) $$.
  - We explored four different covariance structures among these cluster-level variables:
    i. No correlation among any of the covariates
    ii. Compound symmetry such that the off-diagonal correlation coefficients *ρ*_12_ = *ρ*_13_ = *ρ*_23_ = 0.3
    iii. One large correlation between two of the variables (*ρ*_12_ = 0.5, *ρ*_13_ = *ρ*_23_ = 0)
    iv. A correlation structure similar to that observed in the case study dataset: *ρ*_12_ = 0.12, *ρ*_13_ = 0.67, *ρ*_23_ =  − 0.09.
2. Simulate data from three arms under the assumptions specified in step 1. We simulated under simple random allocation in general, assuming **μ** = (0, 0, 0)′ for each arm. We further explored the performance of imbalance metrics via imposing imbalance across arms on average in addition to skewness in select variables. Although it is unrealistic to assume imbalance across arms on average, we sought to determine the performance of the metrics explored under extreme scenarios. Therefore, we explored the following:
  - Balance on average: **μ** = (0, 0, 0)′ for each arm
  - Large imbalance on average: **μ** = (1, 0, 0)′ for arm 1; **μ** = (0, 0, 2) for arm 2; and **μ** = (0, 0, 0)′ for arm 3
  - Slight imbalance on average: **μ** = (1, 0, 0)′ for arm 1 and **μ** = (0, 0, 0)′ for arms 2 and 3
  - Slight imbalance as in 2c but with an added skewness to each variable in each arm
3. Calculate all imbalance metrics for each simulated C-RCT:
  - Minimum of three analysis of variance (ANOVA) *P* values for the statistical test comparing mean of each variable across the three arms [denoted min(ANOVA)]
  - Min(KW three *P* values) as above
  - Multivariate analysis of variable (MANOVA) *P* value for overall test comparing simultaneous means across arms (single *P* value)
  - Minimum of a series of two-sample independent *t* test *P* values (nine total)
    i. Comparing mean of X1, X2, and X3 across arms 1 and 2
    ii. Comparing mean of X1, X2, and X3 across arms 1 and 3
    iii. Comparing mean of X1, X2, and X3 across arms 2 and 3
  - Min(Wilcoxon rank-sum [WRS] test *P* values); nine total, similar to above.

It is important to note that we are using these metrics as tools to evaluate imbalance. We are not using them to test hypotheses in a traditional statistical sense, but we are using them to evaluate how similar or dissimilar distributions in important influential variables may be across randomization arms. In reality, there is no clear definition or gold standard that one may use to state that arms are “balanced” or “imbalanced”, but these metrics are used for relative comparisons.

### Simulation study 1

Initially, we simulated according to a three-arm, equal-allocation design with 10 sites per arm or 30 total sites (10:10:10). We anticipated that this design would be more commonly implemented in cluster-randomized settings than our design involving unequal (1:3:3) allocation. In these simulations, we sought to explore the impact of correlation structure and imposed imbalance and skewness in data. We simulated each scenario above 10,000 times; thus, we ended with (10,000 iterations) × (four correlation structures) × (four mean vector assumptions) = 160,000 simulated C-RCTs with varying levels of (im)balance in three baseline variables across three arms.

### Simulation study 2

Following these initial simulations, we explored the performance of these metrics under a scenario similar to our case study: assuming balance on average or a simple randomized design where **μ** = (0, 0, 0)′ for each arm and unequal allocation (1:3:3): six control sites, 18 sites in intervention arm 1, and 18 in intervention arm 2 for a total of 42 sites. We used correlation structure iv from step 1b above to mimic the structure observed in this dataset. We simulated 100,000 C-RCTs on the basis of these assumptions.

### Simulation study 3

Finally, we resampled from the actual MB study data; the basic logic mirrored that above without distributional assumptions. From the sites with available data, we (a) sampled 10 (without replacement) sites for each arm in each iteration and (b) also sampled under the unequal-allocation scenario as in our present study (6:18:18). Note that we ended with 38 active sites in the current study, but we had available data at baseline for 45 sites total, as several sites dropped out prior to or during the randomization process. We used all available data for resampling described here. For each simulated iteration, we proceeded with step 3 above. We repeated the resampling process 100,000 times for each allocation ratio; that is, we ended with 200,000 simulated C-RCTs with varying levels of (im)balance across three arms in the three actual trial variables.

### Analysis of simulated data

For each simulated trial, we had five metrics of imbalance—all were *P* values corresponding to specific statistical tests. We recognize the flexibility and breadth of possibilities for these imbalance metrics (i.e., we could have chosen to use the test statistics themselves or some other metric). The purpose of these simulations, however, was to explore operating characteristics of our criterion for adequate balance in the true MB study, min(KW *P* value) > 0.30, in comparison with other intuitive measures that we may have otherwise chosen. For each imbalance metric in each simulated C-RCT, we created a dichotomous variable for “adequate” versus “inadequate” balance on the basis of *P* > 0.30. Then we used simple descriptive statistics to explore the sensitivity and specificity of each criterion. The cutoff of *P* > 0.30 to indicate sufficient balance comes from the individually randomized trial literature [9]. Though not explicitly stated by Zhao et al. [9] as a formal recommendation, the *P* > 0.30 may be viewed as “sufficient” and would ensure that our pool of acceptable randomization schemes is not overly restrictive (i.e., that we have a sufficient number of possible randomization sequences in order to prevent bias via over-restriction on the randomization space).

Scenarios simulated under balance on average have the potential to result in chance non-trivial levels of imbalance; similarly, scenarios simulated under imbalance have the potential to result in chance levels of balance. Therefore, we cannot use the rate of adequacy alone to determine the sensitivity and specificity to guide selection of appropriate metrics of imbalance. As we mention above, there is no clear definition or gold standard that one may use to state that arms are “balanced” or “imbalanced”. Thus, we created a new variable: max(mean differences), the standardized (on the standard deviation unit scale) absolute value of the maximum mean difference in any one variable across any two arms. Max(mean difference) > 1.0 may be deemed “unacceptable” or “large” [16] for our purposes, as it would indicate that at least one variable exhibits an entire standard deviation unit difference across two arms. This is a situation that, in a real-world C-RCT setting, one would hope to avoid. We explored the distribution of max(mean difference) and the frequency by which adequate balance by each *P* > 0.30 criterion would result in max(mean difference) > 1.0. Note that, in this case, sensitivity is preferred over specificity, as “conservative” is ideal in terms of controlling imbalance.

## Results

The correlation structure simulated had minimal overall impact; the following results thus collapse all simulated C-RCTs under differing mean vector assumptions into a single scenario for ease of interpretation.

The *t* test and WRS test *P* value criteria tended to be the most conservative in detecting baseline variable imbalance. The MANOVA and ANOVA criteria tended to be least conservative. Recall that, in this context, the term “conservative” refers to a metric that is less likely to accept an individual allocation scenario into the pool of possible scenarios (i.e., one that is more restrictive or constrained). Table 1 illustrates the adequacy rate for each metric—based on the *P* value > 0.30 criterion—for both simulations mirroring our case study at the 1:3:3 allocation ratio: (simulation study 2) the hypothetical data according to a multivariate normal distribution and (simulation study 3) the resampled data. Note that, under simple random allocation, all 100,000 iterations would be deemed adequate; however, we see in Table 1 that only 10%–13% of these scenarios would be deemed adequate for implementation according to the pairwise comparisons involving either the *t* statistic or WRS. Of the metrics explored, the overall MANOVA metric, perhaps unsurprisingly, was the least sensitive, as about 70% of these scenarios were deemed appropriate for implementation. Additional file 1: Table S1 contains similar results for the scenarios exploring imbalance and skewness (simulation study 1). Briefly, when simulating large and unrealistic imbalance on average, all metrics except for the ANOVA and MANOVA deemed all simulated trials inadequately balanced; however, when simulating minor imbalance or skewness (or both), the pairwise tests remained highly sensitive, as over 93% of these purposefully flawed scenarios would never be acceptable according to these metrics. In general, the KW metric demonstrated higher sensitivity than both ANOVA and MANOVA in the imbalanced and skewed scenarios (Additional file 1: Table S1) but comparable sensitivity to the ANOVA for the simulations based on our case study (Table 1).

**Table 1:** Threshold summary statistics by simulated scenario and imbalance criterion (1:3:3 scenarios)

Imbalance criterion	Resampled		Hypothetical	
	N	%	N	%
Min(KW *P* value)	60,508	60.51	62,341	62.34
Inadequate (*P* <0.30)				
Adequate (*P* >0.30)	39,492	39.49	37,659	37.66
min(ANOVA *P* value)	61,691	61.69	61,114	61.11
Inadequate (*P* <0.30)				
Adequate (*P* >0.30)	38,309	38.31	38,886	38.89
MANOVA *P* value	29,997	30.00	29,824	29.82
Inadequate (*P* <0.30)				
Adequate (*P* >0.30)	70,003	70.00	70,176	70.18
Min(*t* test *P* value)	90,223	90.22	87,872	87.87
Inadequate (*P* <0.30)				
Adequate (*P* >0.30)	9777	9.78	12,128	12.13
Min(WRS *P* value)	88,029	88.03	87,114	87.11
Inadequate (*P* <0.30)				
Adequate (*P* >0.30)	11,971	11.97	12,886	12.89

*Abbreviations*: *ANOVA* analysis of variance, *KW* Kruskal–Wallis, *MANOVA* multivariate analysis of variance, *WRS* Wilcoxon rank-sum

**Fig. 2:**
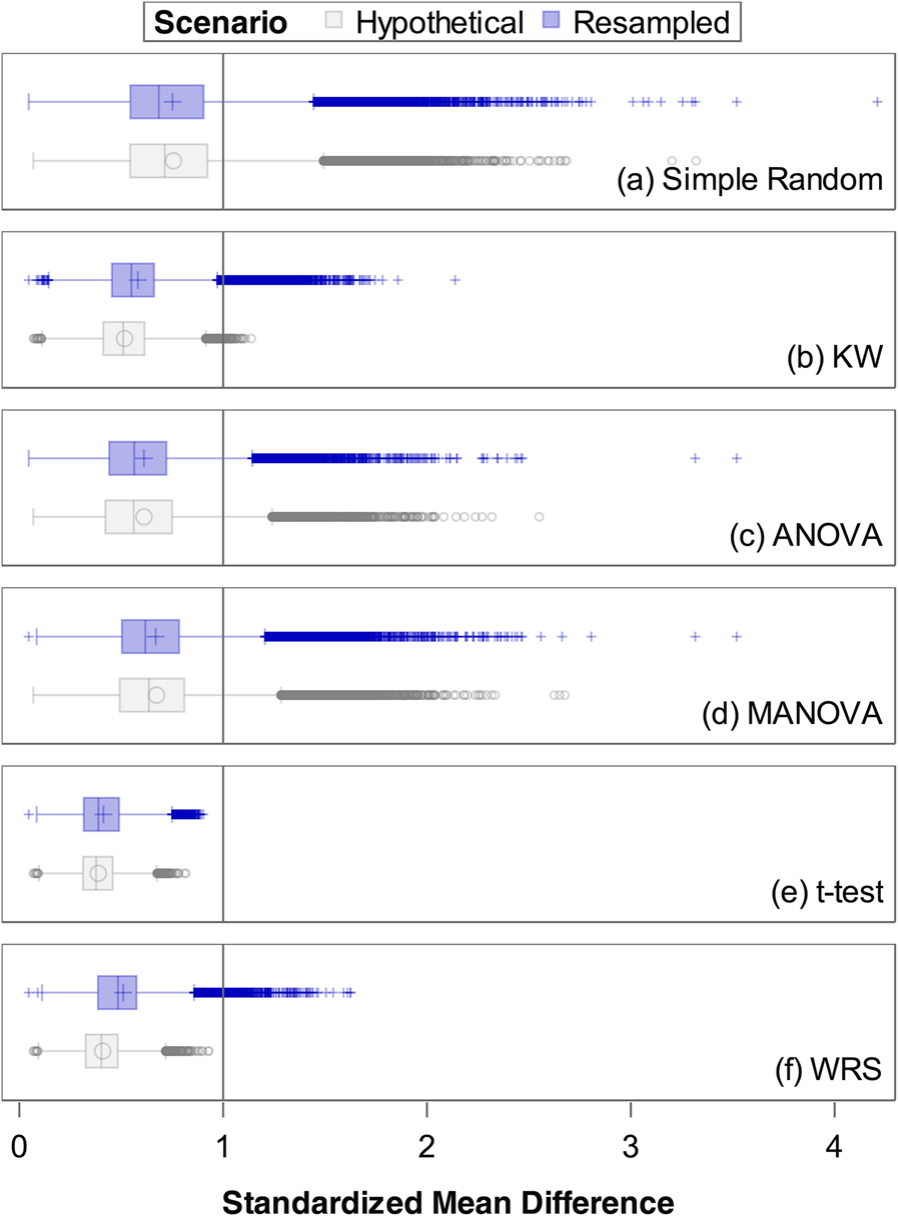
Maximum pairwise imbalance observed for scenarios meeting adequacy threshold (*P* > 0.30) in simulated trials by criterion. Each panel represents the distribution of the max(mean difference) for simulated scenarios meeting the criterion for “adequate” overall variable balance across arms. All simulated schemes meet criteria for adequate under simple random allocation (panel a), but the remaining panels illustrate only those allocation schemes meeting the *P* > 0.30 criterion for each metric. The mean difference depicted is on the standard deviation unit scale. Those meeting this criterion would ideally have a small max(mean difference), and we deem a max(mean difference) > 1.0 (vertical line) unacceptable since previously a value of 0.8 would be deemed “large” [16]

For those scenarios deemed adequate on the basis of the *P* value > 0.30 criterion, Figure 2 shows the distribution of the standardized max(mean difference) variable for the simulations based on the actual study data with unequal allocation (studies 2 and 3). The MANOVA and ANOVA metrics using the 0.30 threshold perform similarly to the simple random allocation scenarios with maximum values at 3.52 for the resampled scenarios and 90th percentiles at 1.00 and 0.94, respectively. The maximum value under simple random allocation was 4.21 with a 90th percentile of 1.16. The pairwise comparisons again demonstrated the most sensitivity, as these metrics almost never allowed implementation of an allocation scheme with max(mean difference) > 1.0. The KW criterion allowed for max(mean difference) as large as 2.14 with a 90th percentile equal to 0.84.

Table 2 presents the sensitivity and specificity of each imbalance criterion in detecting a large (i.e., >1.0) max(mean difference) between any two arms. Sensitivity of the *t* test was 100% for both hypothetical and resampled simulated scenarios, and the WRS demonstrated over 97% in each scenario. The KW criterion maintained higher sensitivity than both the MANOVA and ANOVA criteria (89% and over 99% in the resampled and hypothetical scenarios, respectively) but was not as sensitive as the pairwise criteria. Additional file 1: Table S2 illustrates sensitivities in detecting large levels of pairwise imbalance under the purposefully imbalanced and skewed hypothetical scenarios (study 1). In these instances (assuming equal allocation across 30 sites), the sensitivity of the KW test criterion ranged from 98% to 100%, and the ANOVA and MANOVA criteria exhibited decreased sensitivity for detecting imbalances of 1.0 standard deviation unit mean differences between two arms. In the minor imbalance-on-average scenario, the sensitivities were 65% and 30%, respectively. In the largely imbalanced scenarios, the sensitivities were more than 99% for these two metrics of imbalance.

**Table 2:** Sensitivity and specificity of detecting 1.0 standard deviation max(mean differences) across arms (1:3:3 allocation)

Imbalance criterion	Resampled				Hypothetical			
	max(mean diff) < 1.0		max(mean diff) > 1.0		max(mean diff) < 1.0		max(mean diff) > 1.0	
	N	%	N	%	N	%	N	%
KW	44,340	54.15	16,168	89.27	43,800	53.80	18,541	99.73
Inadequate (*P* <0.30)								
Adequate (*P* >0.30)	37,549	45.85	1943	10.73	37,609	46.20	50	0.27
ANOVA	46,555	56.85	15,136	83.57	45,758	56.21	15,356	82.60
Inadequate (*P* <0.30)								
Adequate (*P* >0.30)	35,334	43.15	2975	16.43	35,651	43.79	3235	17.40
MANOVA	18,933	23.12	11,064	61.09	18,630	22.88	11,194	60.21
Inadequate (*P* <0.30)								
Adequate (*P* >0.30)	62,956	76.88	7047	38.91	62,779	77.12	7397	39.79
*t* test	72,112	88.06	18,111	100.00	69,281	85.10	18,591	100.00
Inadequate (*P* <0.30)								
Adequate (*P* >0.30)	9777	11.94	0	0	12,128	14.90	0	0
WRS	70,287	85.83	17,742	97.96	68,523	84.17	18,591	100.00
Inadequate (*P* <0.30)								
Adequate (*P* >0.30)	11,602	14.17	369	2.04	12,886	15.83	0	0

*Abbreviations*: *ANOVA* analysis of variance, *KW* Kruskal–Wallis, *MANOVA* multivariate analysis of variance, *WRS* Wilcoxon rank-sum

**Fig. 3:**
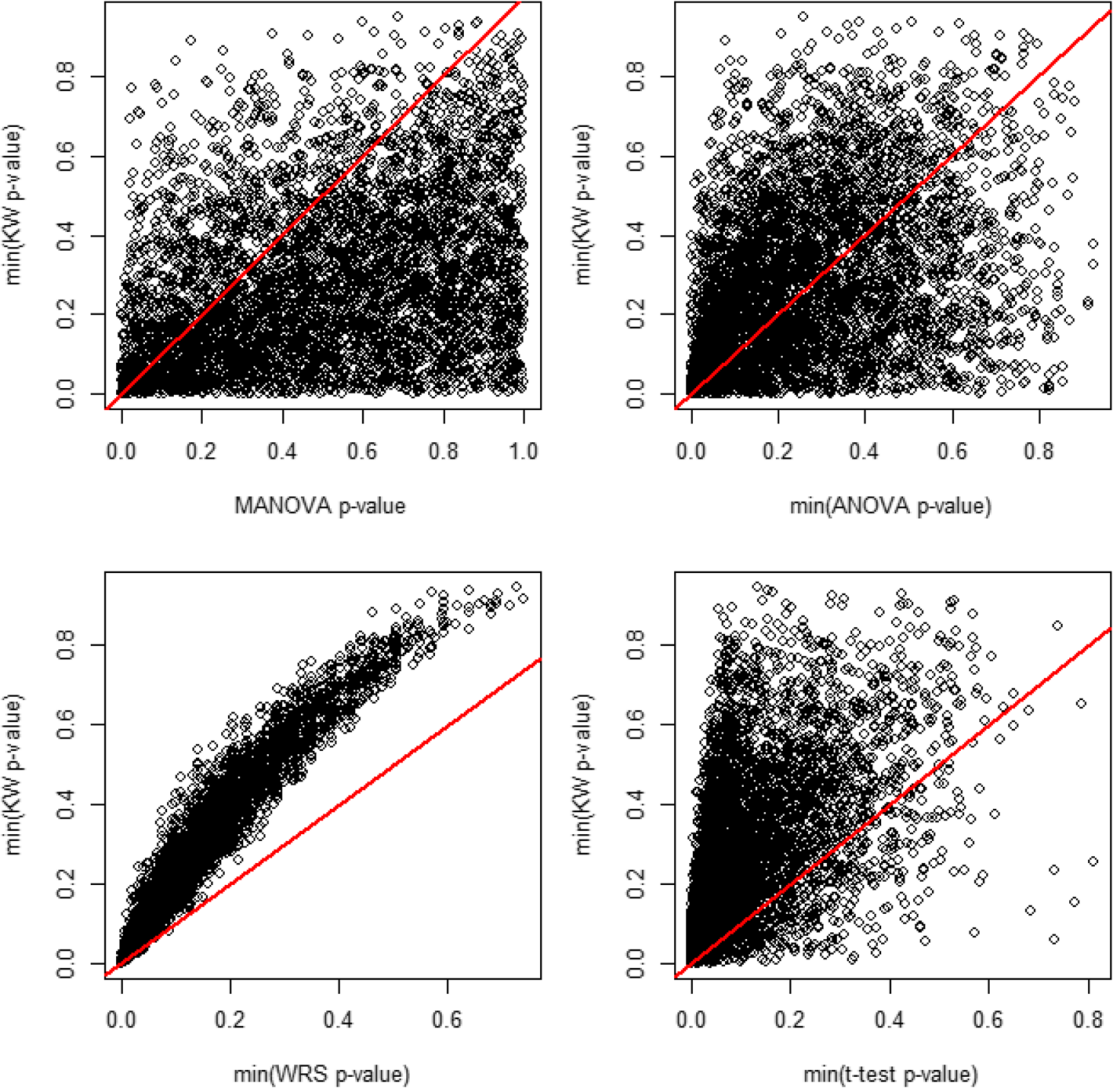
Pairwise scatterplots exploring associations between Kruskal–Wallis (KW) *P* value with other measures. The panels here present a selection of pairwise plots to illustrate the relationships between the imbalance metric we used in our randomization algorithm, the KW test *P* value, and additional candidate imbalance metrics explored. Each plot includes 5000 observations from the resampled scenarios using 1:3:3 allocation as in our present study. In each plot, there is often a non-linear relationship. For example, the first plot illustrating min(KW *P* value) in comparison with the multivariate analysis of variance (MANOVA) demonstrates a somewhat noisy relationship between the two whereby the min(KW *P* value) tends to be lower than the overall MANOVA *P* value, but the two are related. The comparison of the min(KW *P* value) versus the min(Wilcoxon rank-sum [WRS] *P* value) shows a more pronounced relationship and a clearer, non-linear pattern. All metrics of imbalance as determined are highly related; broadly, the more global tests (e.g., MANOVA) tended to be less conservative (i.e., have larger *P* value) than the corresponding more specific tests based on more than one comparison (e.g., WRS). The line y = x has been added for reference

For each test *P* value, we chose a criterion of *P* value > 0.30 to signify adequate balance. Although we anticipate tests’ *P* values to be correlated with one another, they will not exhibit a linear one-to-one relationship. We would expect the *P* values exploring difference in any individual variable across two arms (i.e., the *t* test and WRS *P* values) to be more sensitive than those evaluating an individual variable across all three arms (i.e., the KW and ANOVA *P* values); in turn, we would expect the global test (MANOVA) to demonstrate the least sensitivity. Here, we determine sensitivity on the basis of the distribution of these *P* values. Figure 3 illustrates a series of scatterplots comparing the KW *P* value with each additional metric explored in these simulations.

## Discussion

The case study presented here illustrates some complexities that may arise in a real-world C-RCT setting. Under simple random allocation (i.e., when we simulated balance on average), we observed non-trivial (>1.0 standard deviation unit mean difference across two arms) levels of imbalance nearly 20% of the time in these when simulating data similar to those seen in our case study. Thus, we would like to be able to detect and prevent such levels of imbalance at baseline in an actual C-RCT. Modified constrained randomization procedures allow us to determine a subset of adequately balanced intervention allocation schemes, but we illustrate the care that one must take in choosing an imbalance metric for continuous baseline variables across multiple arms.

When we have more than two arms, typical metrics [6, 12, 13] become difficult to use, as they often focus on categorical variables or those readily applied to the two-arm, equal-allocation scenario. In our present study, we chose the min(KW test *P* value) > 0.30 to signify adequate imbalance. We made this decision piecing together intuition and the guidance in the literature at the time. However, we present simulations here that illustrate other candidate measures of imbalance that have varying abilities to detect large imbalance under many scenarios. It is not surprising that some metrics are more conservative than others. We would expect those evaluating on all possible pairwise differences (i.e., the *t* test and WRS criteria) to be more conservative and have the ability to detect levels of imbalance much smaller than the global metrics (i.e., the MANOVA). Recall that "conserative" in this sense refers to a metric that is more likely to indicate imbalance or result in fewer possible allocation schemes that would be deemed acceptable. We chose the min (KW test *P* value) because of the small number of sites in our study across three arms and the anticipated violation of the normality assumptions for each of the three actual study variables.

The simulation results give us confidence that our chosen KW test-based metric implemented in our study (*P* > 0.30) is robust and has high sensitivity in general for detecting non-trivial levels of baseline covariate imbalance. The MANOVA and ANOVA test criteria (using *P* > 0.30 for adequate levels of imbalance) were not as sensitive in general. In fact, the distribution of the max(mean difference) for scenarios deemed acceptable according to these criteria looks similar to that of the simple randomization scenarios (Fig. 2). This suggests that using one of these metrics to indicate sufficient levels of balance as part of a modified CCR scheme has a performance similar to that of simple randomization.

The *t* test and WRS test *P* value > 0.30 criteria were the most conservative the most often in all simulated scenarios. Using one of these metrics would likely also ensure adequate baseline covariate balance in similar C-RCTs. One potential drawback, however, may be a problem of potential over-constraint; specifically, the recommendation for analyses based on randomization through permutation tests relies heavily on the constrained randomization space [11, 17]. For example, when we examine the simulated scenarios imposing unrealistic but minor imbalance on average, the *t* test and WRS metrics would deem these scenarios adequate just 3–4% of the time. This may not matter if analyses do not call for permutation tests. However, use of these metrics makes a permutation test less plausible.

### Study limitations and alternative approaches

We recognize the fact that we have explored a finite number of imbalance metrics options when, in reality, there are an infinite number of metrics we could have chosen. We originally chose the KW test *P* value criterion as it is intuitive and easy to use. The others explored in these simulations are also intuitive and fairly easy to use. The cutoff of *P* > 0.30 to indicate sufficient balance stemmed from the recommendation of Zhao et al. in individually randomized trial literature [9]. As previously mentioned, another suggestion from the C-RCT literature involves using the 10th percentile (upper/lower, depending upon the measure) of all simulated/enumerated allocations to determine the pool of “acceptable” allocation schemes [6, 11]. Although we could have adopted this criterion in our current trial or in the simulations, we found this notion not as intuitive. In fact, the corresponding upper/lower thresholds depend highly on the underlying distribution of variables across arms, although in theory the underlying assumption is that the arms are balanced on average. When we resampled the MB data in our simulations, the 90th percentile *P* values for MANOVA, ANOVA, KW, *t* test, and WRS test were 0.90, 0.57, 0.59, 0.30, and 0.32, respectively. The lower 10th percentile of the maximum mean difference across arms was 0.43. If data were to come from a theoretically skewed or imbalanced distribution or both (although this is very unlikely in a real C-RCT setting), these thresholds would be different. Therefore, using the KW test *P* value > 0.30 criterion that we chose allows a larger pool of possible treatment allocation schemes that otherwise would have been thrown out, as they would not have met the 90th percentile criterion.

Finally, a note should be made regarding the analyses of such C-RCTs. Recently, Turner et al. presented a review of design [2] analysis methods for C-RCTs [17]. As mentioned in section 2, the unit of analyses for this MB case study is the individual participant. Therefore, the site-level variables we explore here are only surrogates for the true underlying participant-level variables (i.e., race, environment, and level of individualized care) for which we ultimately hope to control imbalance as a result of the randomization scheme implemented. Thus, we cannot be sure whether our algorithm was truly successful in achieving relative balance across arms until we assess final participant-level variables for all enrolled participants; this research is under way. There may be some unmeasured or unaccounted for variables that can present bias in analyses, but the analytic strategy will account for intra-class correlation and important participant-level covariates appropriately as recommended in analyses of C-RCTs [17, 18] in order to minimize type I error rate inflation, bias, and ultimately false conclusions.

## Conclusions

Although any of the metrics we explore here for ensuring comparability in C-RCTs may suffice for an individual trial, we use these simulations as a guide to researchers who are planning to implement C-RCTs with CCR techniques. To researchers planning such a trial, especially one with more than two arms, we suggest the following:

1. Always consider baseline variables in both the design and analysis phase in C-RCTs; we cannot assume that simple randomization will solve all problems with respect to covariate imbalance [7, 8, 19].
2. Consider using the KW test *P* value > 0.30 as in our scenario, as it seemed sensitive and not overly conservative.
3. Consider using continuous variables in place of categorical variables because of the potential sparsity in cell counts. It is worth noting that the choice of a cut point or threshold for variables otherwise considered continuous may be somewhat arbitrary and come at a cost as well [20].
4. Explore properties of imbalance metrics prior to implementation in any real C-RCT. Any of the imbalance metrics we explore here are potential candidate measures, but care should be taken to determine the appropriate threshold for “adequate” balance.
5. Other metrics to consider include the following:
  - The MANOVA or ANOVA with larger threshold *P* value (e.g., simulations suggest ANOVA *P* >0.56 and MANOVA *P* >0.90) that corresponds to the upper 10% as recommended by Raab and Butcher [6].
  - The pairwise tests (i.e., *t* test and WRS test), although these have potential to be overly conservative. If the research does not plan to use permutation tests or if weights imbalance control more heavily than overly constrained randomization, these tests may be the better option.

## Additional file

Additional file 1: **Table S1.** Threshold summary statistics by simulated scenario and imbalance criterion (1:1:1 Scenarios). **Table S2.** Sensitivity and specificity of detecting 1.0 standard deviation max(mean differences) across arms (1:1:1 allocation). (DOCX 25 kb)

## Abbreviations

ANOVA: Analysis of variance
CCR: Covariate-constrained randomization
C-RCT: Cluster-randomized controlled trial
HV: Home visitor
KW: Kruskal–Wallis
MANOVA: Multivariate analysis of variance
MB: Mothers and Babies
MH: Mental health
WRS: Wilcoxon rank-sum
