# Supplementary material for: Choosing an imbalance metric for covariate-constrained randomization in multiple-arm cluster-randomized trials
Source: Trials. 2019 May 28;20:293. doi: 10.1186/s13063-019-3324-5 (PMC6537428; doi:10.1186/s13063-019-3324-5)
Supplement: Supplementary file 1 — Table S1. Threshold summary statistics by simulated scenario and imbalance criterion (1:1:1 Scenarios). Table S2. Sensitivity and specificity of detecting 1.0 standard deviation max(mean differences) across arms (1:1:1 allocation). (DOCX 25 kb) [file 13063_2019_3324_MOESM1_ESM.docx]

**Table S1. Threshold Summary Statistics by Simulated Scenario and Imbalance Criterion (1:1:1 Scenarios)**

| IMBALANCE CRITERION | Balance on Average | | Minor Imbalance | | Large Imbalance | | Skewed + Minor Imbalance | | Resampled | |
| --- | --- | --- | --- | --- | --- | --- | --- | --- | --- | --- |
|  | N | % | N | % | N | % | N | % | N | % |
| min(KW p-value) | 25543 | 63.86 | 37820 | 94.55 | 40000 | 100.00 | 30804 | 77.01 | 60310 | 60.31 |
| Inadequate (p<0.30) |  |  |  |  |  |  |  |  |  |  |
| Adequate (p>0.30) | 14457 | 36.14 | 2180 | 5.45 | 0 | 0 | 9196 | 22.99 | 39690 | 39.69 |
| min(ANOVA p-value) | 25318 | 63.30 | 24393 | 60.98 | 39989 | 99.97 | 26232 | 65.58 | 62566 | 62.57 |
| Inadequate (p<0.30) |  |  |  |  |  |  |  |  |  |  |
| Adequate (p>0.30) | 14682 | 36.71 | 15607 | 39.02 | 11 | 0.03 | 13768 | 34.42 | 37434 | 37.43 |
| MANOVA p-value | 11945 | 29.86 | 10449 | 26.12 | 39863 | 99.66 | 11910 | 29.78 | 30345 | 30.35 |
| Inadequate (p<0.30) |  |  |  |  |  |  |  |  |  |  |
| Adequate (p>0.30) | 28055 | 70.14 | 29551 | 73.88 | 137 | 0.34 | 28090 | 70.23 | 69655 | 69.66 |
| MIN(T-test P-value) | 35800 | 89.50 | 39736 | 99.34 | 40000 | 100.00 | 37666 | 94.17 | 89689 | 89.69 |
| Inadequate (p<0.30) |  |  |  |  |  |  |  |  |  |  |
| Adequate (p>0.30) | 4200 | 10.50 | 264 | 0.66 | 0 | 0 | 2334 | 5.84 | 10311 | 10.31 |
| MIN(WRS P-value) | 35282 | 88.21 | 39616 | 99.04 | 40000 | 100.00 | 37558 | 93.90 | 88280 | 88.28 |
| Inadequate (p<0.30) |  |  |  |  |  |  |  |  |  |  |
| Adequate (p>0.30) | 4718 | 11.80 | 384 | 0.96 | 0 | 0 | 2442 | 6.11 | 11720 | 11.72 |

**Table S2. Sensitivity and Specificity of Detecting 1.0 Standard Deviation Max(Mean Differences) across Arms (1:1:1 Allocation)**

| IMBALANCE CRITERION | Balance on Average | | | | Minor Imbalance | | | | Large Imbalance | | | | Skewed + Minor Imbalance | | | | Resampled | | | |
| --- | --- | --- | --- | --- | --- | --- | --- | --- | --- | --- | --- | --- | --- | --- | --- | --- | --- | --- | --- | --- |
|  | max(mean diff)<1.0 | | max(mean diff)>1.0 | | max(mean diff)<1.0 | | max(mean diff)>1.0 | | max(mean diff)<1.0 | | max(mean diff)>1.0 | | max(mean diff)<1.0 | | max(mean diff)>1.0 | | max(mean diff)<1.0 | | max(mean diff)>1.0 | |
|  | N | % | N | % | N | % | N | % | N | % | N | % | N | % | N | % | N | % | N | % |
| KW | 15629 | 51.95 | 9914 | 99.99 | 8307 | 79.22 | 29513 | 100.00 | 22 | 100.00 | 39978 | 100.00 | 17038 | 64.96 | 13766 | 99.96 | 48093 | 54.93 | 12217 | 98.10 |
| Inadequate (p<0.30) |  |  |  |  |  |  |  |  |  |  |  |  |  |  |  |  |  |  |  |  |
| Adequate (p>0.30) | 14456 | 48.05 | 1 | 0.01 | 2179 | 20.78 | 1 | 0.00 | 0 | 0 | 0 | 0 | 9191 | 35.04 | 5 | 0.04 | 39453 | 45.07 | 237 | 1.90 |
| ANOVA | 17018 | 56.57 | 8300 | 83.71 | 5180 | 49.40 | 19213 | 65.10 | 22 | 100.00 | 39967 | 99.97 | 15461 | 58.95 | 10771 | 78.22 | 51929 | 59.32 | 10637 | 85.41 |
| Inadequate (p<0.30) |  |  |  |  |  |  |  |  |  |  |  |  |  |  |  |  |  |  |  |  |
| Adequate (p>0.30) | 13067 | 43.43 | 1615 | 16.29 | 5306 | 50.60 | 10301 | 34.90 | 0 | 0 | 11 | 0.03 | 10768 | 41.05 | 3000 | 21.78 | 35617 | 40.68 | 1817 | 14.59 |
| MANOVA | 5982 | 19.88 | 5963 | 60.14 | 1498 | 14.29 | 8951 | 30.33 | 18 | 81.82 | 39845 | 99.67 | 5158 | 19.67 | 6752 | 49.03 | 22056 | 25.19 | 8289 | 66.56 |
| Inadequate (p<0.30) |  |  |  |  |  |  |  |  |  |  |  |  |  |  |  |  |  |  |  |  |
| Adequate (p>0.30) | 24103 | 80.12 | 3952 | 39.86 | 8988 | 85.71 | 20563 | 69.67 | 4 | 18.18 | 133 | 0.33 | 21071 | 80.33 | 7019 | 50.97 | 65490 | 74.81 | 4165 | 33.44 |
| t-test | 25885 | 86.04 | 9915 | 100.00 | 10222 | 97.48 | 29514 | 100.00 | 22 | 100.00 | 39978 | 100.00 | 23895 | 91.10 | 13771 | 100.00 | 77235 | 88.22 | 12454 | 100.00 |
| Inadequate (p<0.30) |  |  |  |  |  |  |  |  |  |  |  |  |  |  |  |  |  |  |  |  |
| Adequate (p>0.30) | 4200 | 13.96 | 0 | 0 | 264 | 2.52 | 0 | 0 | 0 | 0 | 0 | 0 | 2334 | 8.90 | 0 | 0 | 10311 | 11.78 | 0 | 0 |
| WRS | 25367 | 84.32 | 9915 | 100.00 | 10102 | 96.34 | 29514 | 100.00 | 22 | 100.00 | 39978 | 100.00 | 23787 | 90.69 | 13771 | 100.00 | 75854 | 86.64 | 12426 | 99.78 |
| Inadequate (p<0.30) |  |  |  |  |  |  |  |  |  |  |  |  |  |  |  |  |  |  |  |  |
| Adequate (p>0.30) | 4718 | 15.68 | 0 | 0 | 384 | 3.66 | 0 | 0 | 0 | 0 | 0 | 0 | 2442 | 9.31 | 0 | 0 | 11692 | 13.36 | 28 | 0.22 |
